# Investigating Falls Risk Awareness in Hospitals Using the Self‐Awareness of Falls Risk Measure (SAFRM): Empirical Research Quantitative

**DOI:** 10.1002/nop2.70099

**Published:** 2024-12-26

**Authors:** Elissa Dabkowski, Simon J. Cooper, Jhodie Duncan, Karen Missen

**Affiliations:** ^1^ Institute of Health and Wellbeing Federation University Australia Churchill Victoria Australia; ^2^ School of Nursing and Midwifery Edith Cowan University Perth Western Australia Australia; ^3^ Latrobe Regional Health Traralgon West Victoria Australia

**Keywords:** fall prevention, falls, falls risk, hospital, patient, perception

## Abstract

**Aim:**

The overarching aim of this study was to explore patients' falls risk awareness in hospitals using section A of the validated Self Awareness of Falls Risk Measure (SAFRM).

**Design:**

Descriptive cross‐sectional study design.

**Setting:**

Three rural/regional hospitals in the State of Victoria, Australia.

**Methods:**

Using a purposive sampling strategy, patients were eligible to participate if aged ≥ 40 years, English‐speaking, and have ambulatory capacity prior to hospital admission. Participants were excluded from the study if they returned a Standardised Mini‐Mental State Examination (SMMSE) score < 18. Falls risk awareness data was collected from both patient and health professionals using section A of the SAFRM. Patient demographic data was collected from patient medical records.

**Results:**

A total of 77 patients (72.9 years ±11.2) and 58 health professionals were recruited. Patients had a significant difference in falls risk awareness when compared to their clinician (*z* = −2.08, *p* = 0.038). Regression analyses showed that patients were more likely to overestimate their falls risk if they used anticoagulant medication and if their highest education level was less than or equal year 11. An exploratory factor analysis (EFA) revealed a three‐factor solution from section A of the SAFRM, which were labelled Physical Activity Awareness, Cognitive Awareness and Balance Awareness.

**Conclusions:**

There was a significant difference in patients' falls risk awareness compared to a health professional. The independent associations of variables with falls risk awareness, such as age, education level and medication use, further our understanding of the differences in falls risk awareness. The findings also establish that the 15‐item section A SAFRM is a reliable and feasible falls risk perception measure for use in hospitals, with future research recommended to evaluate the proposed three‐factor model with the addition of tailored hospital falls education.

**Implications for the Profession and/or Patient Care:**

The findings from this study establish a significant difference in patients' falls risk awareness compared to their health professional. Section A of the SAFRM is a reliable tool for nurses and other health professionals to establish the presence of a falls risk disparity. The ability to empirically measure this disparity and to determine an under‐ or overestimation of falls risk is a useful addition to clinical practice. The SAFRM facilitates a person‐centred approach to falls prevention by providing opportunities for the clinician to collaborate with the patient and tailor fall prevention strategies.

**Impact:**

*Problem*: Inpatient falls in hospital settings. *Main Findings*: There was a significant difference in patients' falls risk awareness compared to a health professional. Section A of the Self‐Awareness of Falls Risk measure is a reliable and feasible tool to identify under‐ or overestimation of falls risk perception in hospitals. Patients were more likely to overestimate their falls risk if they used anticoagulant medication and if their highest education level was less than year 11. The findings for a three factor‐model Physical Activity Awareness, Cognitive Awareness and Balance Awareness could inform future hospital falls education. *Impact*: Registered nurses, health professionals, inpatients. *Reporting Method*: STROBE checklist for cross‐sectional studies. *Patient or Public Contribution*: This study involved the collection of data from patient participants and registered nurses.

## 1 Introduction

Falls prevention in hospital continues to be a high concern for healthcare organisations because of the potential for adverse outcomes. It is estimated that approximately 700,000 to 1 million patient falls that occurred in United States of America, resulted in increased patient harm and morbidity (LeLaurin and Shorr 2019). Although falls can occur in all environments, there is an expectation that patients will be safe from harm in a high‐quality health system (Australian Institute of Health and Welfare 2022). In hospitals, the risk for falling is higher due to the unfamiliarity of the environment, co‐morbidities, medication, cognitive impairment, delirium and prolonged weakness (Deandrea et al. 2013; Oliver et al. 2004). However, research shows that many people do not regard themselves to have a high falls risk in hospital (Dabkowski et al. 2022; Dabkowski, Cooper, et al. 2023; Dolan, Slebodnik, and Taylor‐Piliae 2021; Heng et al. 2020). This is important because falls risk awareness disparities, low self‐efficacy and reduced compliance with fall prevention strategies can contribute to falls in hospitalised adults (Mihaljcic et al. 2017). Understanding patients' falls risk awareness may help to inform falls management strategies in hospital.

## 2 Background

The importance of establishing patients' falls risk awareness has gained momentum in contemporary literature. Recent World Guidelines for Falls Prevention and Management strongly recommended incorporating the perspective of the older adult, as part of a multifactorial risk assessment to inform decision‐making on interventions (Montero‐Odasso et al. 2022). These recommendations included the use of a standardised instrument to assess the patient's concerns about falling in acute care hospitals or long‐term care facilities (Montero‐Odasso et al. 2022). Patient Reported Outcome Measures (PROMs) are examples of such measures, which can be used by clinicians to establish patients' perceptions of their symptoms and their physical functioning (Black 2013). Previous literature has established the existence of several fall risk psychological measures, which are often used interchangeably to measure psychological constructs (Jørstad et al. 2005; Moore and Ellis 2008). Researchers are encouraged to report and classify the falls‐related psychological constructs under observation to promote transparency (Hughes et al. 2015; Moore and Ellis 2008) A recent Consensus‐based Standards for the selection of health Measurement Instruments (COSMIN) systematic review reported a total of 20 falls risk perception measures validated for use in a hospital setting (Dabkowski, Missen, et al. 2023). These falls risk perception measures were defined under the following constructs: Balance Confidence, Fall‐related Self‐Efficacy, Fear of Falling, Falls Risk Awareness and Outcome Expectancy (Dabkowski, Missen, et al. 2023). The inclusion of Falls Risk Awareness as a falls‐related psychological construct builds upon previous findings from Hughes et al. (2015) and Moore and Ellis (2008). In this context, Falls Risk Awareness relates to the person's understanding of their personal strengths and limitations (Caldwell and Hayes 2016; Dabkowski, Missen, et al. 2023).

Of the 20 falls risk perception instruments in the COSMIN systematic review, five PROMs were classified under the Falls Risk Awareness construct. Only one instrument, the Self Awareness of Falls Risk Measure (SAFRM), measured Falls Risk Awareness from the perspective of both the patient and clinician (Dabkowski, Missen, et al. 2023). Using the COSMIN guidelines, the SAFRM received a Class B recommendation, which indicates the PROM has the potential to be recommended for use in practice but requires further research to assess its quality (Mokkink et al. 2018).

The SAFRM was developed to assess self‐awareness of falls risk in an inpatient rehabilitation setting compared to that of a health professional (Mihaljcic et al. 2014). The theoretical foundation of this measure was conceptualised using the Self‐Awareness Model from the head injury literature. The Self‐Awareness Model was originally developed by Crosson et al. (1989) and contains three hierarchical levels of awareness. *Intellectual awareness* pertains to the individual's ability to recognise that their physical, behavioural or cognitive function is impaired; *emergent awareness* pertains to the individual's ability to identify a problem as it is occurring and *anticipatory awareness* refers to the individual's ability to anticipate that a problem may occur and may take steps to prevent it. The original SAFRM contains three subsections for a total of 31 scale items and requires completion by both the patient and clinician, as well as a Timed Up‐and‐Go test for subsection two. A reliability analysis was conducted and reported in the original SAFRM for each individual subsection (Mihaljcic et al. 2014). Intellectual awareness, or section A of the SAFRM demonstrated high internal consistency (*α* = 0.89) and high inter‐rater reliability between clinicians (ICC = 0.78) (Mihaljcic et al. 2014). Section B, emergent awareness demonstrated high internal consistency (*α* = 0.90) with moderate inter‐rater reliability amongst clinicians (ICC = 0.61) (Mihaljcic et al. 2014). Whereas anticipatory awareness or section C of the SAFRM also showed high internal consistency (*α* = 0.86) with excellent inter‐rater reliability (*α* = 0.80) (Mihaljcic et al. 2014). Convergent validity of section A of the SAFRM was established (*ρ* = 0.50, *p* < 0.001), as was ecological validity of the clinician ratings (*ρ* = −0.57, *p* < 0.001), which relates to real life settings (Mihaljcic et al. 2014). Given the high internal consistency of section A, the authors were interested in exploring the first 15‐item subsection of the SAFRM to determine its suitability for assessing falls risk awareness. It was anticipated that using section A of the SAFRM would be a feasible option for clinicians whilst still resulting in meaningful patient‐reported data.

By definition, the SAFRM is considered a pragmatic and patient‐centred measure applicable for use in real‐world settings. As such, pragmatic measures are intended to be conducted by various relevant professionals and/or settings (Glasgow and Riley 2013). Using the designated scoring system, clinicians are able to determine if the patient under‐ or overestimated their falls risk compared to the clinician's scores (Mihaljcic et al. 2014). The SAFRM also enables the user to determine the extent of such disparities in falls risk awareness. This instrument has the potential to facilitate and inform individual falls prevention strategies in a hospital setting. Previous studies recommended further investigation of the SAFRM in larger and diverse settings, with prior studies undertaken in metropolitan regions (Mihaljcic et al. 2015, 2017). Therefore, further exploration of the SAFRM in alternative settings may increase understanding of patients' falls risk awareness. This line of questioning is warranted, given the strong recommendations from the World Falls Guidelines about clinicians enquiring about falls' perceptions (Montero‐Odasso et al. 2022).

## 3 Aim

The overarching aim of this study was to explore patients' falls risk awareness in hospitals compared to a health professional using section A of the SAFRM. Specific research objectives for this study are (i) to determine the extent of disparities in patients' falls risk awareness in hospital compared to a treating health professional, (ii) to establish if a relationship exists between inpatients' falls risk disparities and patient characteristics, (iii) to conduct a factor analysis to assess the construct validity of section A of the SAFRM and (iv) to investigate the reliability of utilising section A of the SAFRM to measure falls risk awareness in patients.

## 4 Method

### 4.1 Design

This study follows a descriptive cross‐sectional design to characterise the prevalence of falls risk awareness in a specified population. It was reported using a Strengthening The Reporting of Observational Studies in Epidemiology (STROBE) checklist for cross‐sectional studies (von Elm et al. 2007).

### 4.2 Ethical Considerations

Prior to participant recruitment, high‐risk ethical approval was received from both Latrobe Regional Health Hospital Human Research Ethics Committee and Federation University Human Research Ethics Committee (2022‐02 HREA and 2022‐057 respectively). All participants received a plain language information statement (PLIS) and were instructed they could withdraw from the study at any time prior to data aggregation. Participants were informed both verbally and in the PLIS that any data collected would be de‐identified and assigned a case number to protect their anonymity. Written informed consent was obtained and this process is detailed in the Procedure section.

### 4.3 Setting and Sample

This study was conducted across various wards (acute medical, surgical, orthopaedic and rehabilitation) in three rural/regional hospitals in the State of Victoria, Australia, for a period of 2 months (30 May 2022 to 28 July 2022). The three hospitals consist of a large 289‐bed hospital, a rural 70‐bed acute/subacute hospital and a regional 36 acute/subacute facility. These hospitals were selected based on an unpublished snapshot clinical falls audit that highlighted a problematic inpatients falls issue. An assumption from this study is the patient population from the three hospitals are homogenous in nature due to comparable Socio‐Economic Indexes for Area (SEIFA) scores. Inpatients were recruited using a non‐probabilistic, consecutive sampling strategy, where they were eligible to participate if aged ≥ 40 years, communicated in English and had ambulatory capacity prior to their hospital admission. Ambulatory capacity refers to people who are able to walk including those with a gait aid. Patients were excluded from participating if they had severe cognitive deficits, delirium or an acute psychiatric illness.

A power analysis of the estimated sample size was calculated using the statistical program G*Power (Faul et al. 2007). An *α priori* two‐tailed t‐test was conducted using a medium effect size (*d* = 0.5), 80% power and a 95% confidence level, calculating a projected sample size of 128 (64 patients and 64 health professionals). A sample size estimation was also undertaken for a multiple linear regression using the SAFRM score as the dependent variable and sociodemographic and other variables as predictors. This was calculated to be a total of 55 patients for a medium effect size (*f* ^2^ = 0.15). Given that a sample of 64 patients is required to address the first research objective, this is a sufficient sample size to address the second research objective. To account for potential missing values, an estimated 20% was added to the projected sample size increasing the total number of participants to 154 (77 patients and 77 health professionals). The proposed sample size of 77 patients is comparable to a previous sample size (*n* = 84) that was used to validate section A of the original SAFRM study (Mihaljcic et al. 2014).

### 4.4 Data Collection Tools

#### 4.4.1 Self‐Awareness of Falls Measure (SAFRM)

The 15‐item subsection for intellectual awareness (section A) measures vision, transfer balance, leg strength, steadiness during walking, proprioception, impulsivity, concentration, following instructions, safety decision‐making and various activities of daily living such as showering, toileting and walking on a 5‐point Likert scale (Mihaljcic et al. 2014). The background section contains information about the validity and reliability of this instrument. Email permission was received from the original authors to use the 15‐item intellectual awareness subsection. Please see supplementary file for the full version of the SAFRM.

#### 4.4.2 Clinician‐Rated SAFRM

The self‐awareness of falls risk is calculated by subtracting the clinician‐ratings from the patient‐ratings to determine any awareness deficits in each subsection. Therefore, health professionals such as nursing staff, physiotherapists and occupational therapists, were also recruited to complete the clinician component of section A of the SAFRM. A positive SAFRM score indicated that a person underestimated their falls risk and a negative SAFRM score represented an overestimation of falls risk. A discrepancy score between −10 and 10 indicates complete awareness of falls risk, a score between 11 and 20 indicates mild discrepancy, 21–30 indicates moderate discrepancy and > 30 indicates severe discrepancy (Mihaljcic et al. 2015).

The clinician component contains the same 5‐point Likert scale and questions as the patient version. Eligible health professionals of consenting patients were approached by the lead researcher to invite them to participate in the study. Health professionals were eligible to participate if they had previously assessed the patient and provided care. Health professionals were invited to share their years of clinical experience at the top of the SAFRM.

#### 4.4.3 Cognition

Cognitive functioning was measured using the Standardised Mini‐Mental State Examination (SMMSE), which has an intraclass correlation of 0.90 (Molloy and Standish 1997). A score between 18 and 24 on the SMMSE indicates mild to moderate cognitive impairment (Molloy and Standish 1997). The inclusion of people with mild to moderate cognitive impairment is an important consideration, as this population are at higher risk of falling compared to adults who are cognitively intact (Härlein et al. 2009).

#### 4.4.4 Patient Characteristics

Participant demographics were collected from patients' medical records including age, gender, cultural background, living situation, highest level of education attained, current admission diagnosis, number of medications, medication type, pre‐admission medical history, falls history, Falls Risk Assessment Tool (FRAT) score (as assessed by their treating nurse), gait aid use, area of hospital and the number of days in hospital at time of interview. These variables were selected based on their relevance from studies featured in a scoping review (Dabkowski et al. 2022). This data was iteratively coded into categories (see Appendix S1).

### 4.5 Procedure

The consecutive sampling strategy involved the lead researcher liaising with the assistant nurse unit manager (ANUM) at the start of the shift. The ANUM identified inpatients based on their suitability to the eligibility criteria and used clinical judgement to identify those suspected to obtain a score of ≥ 18 on the SMMSE. The ANUM also communicated which patients were clinically unsuitable for this study for example, such as those in COVID isolation. The lead researcher visited each patient who met the eligibility criteria, explained the study and obtained the appropriate permissions, including written informed consent. Participants had the opportunity to ask questions and were not obligated to provide an immediate response about partaking in the study. After conducting the SMMSE and the section A SAFRM, the lead researcher approached a treating health professional involved in the participant's care and obtained written consent. The health professional completed the clinician component of the section A SAFRM within the same clinical shift. Demographic data was retrospectively obtained by the lead researcher from the patient's clinical records. If a participant scored 17 or lower on the SMMSE, data collection ceased and the ANUM was informed. The collected data was entered into IBM SPSS Statistics (Version 29).

### 4.6 Data Analysis

To establish the significance of any results, paired samples *t*‐tests were used if data met statistical assumptions. If the data violated statistical assumptions, Wilcoxon signed‐ranks test would be used. This test is also known as the Wilcoxon matched pairs signed ranks test and is intended for use with repeated measures or for when participants are measured under two different conditions (Pallant 2011). Statistical significance was established if *p* < 0.05.

The treatment of over‐ and underestimating of falls risk as a continuum may impact the interpretability of results, given they may have different underlying causes (Mihaljcic et al. 2015). Therefore, to conduct the correlational and regression analyses, the two groups of overestimation and underestimation of falls risk were separated. Correlational and regression analyses were conducted to establish if a relationship exists between inpatients' falls risk disparities and patient characteristics. Pearson correlation coefficients were used if the variables met statistical assumptions, whereas Spearman correlation coefficients were used in the event of statistical violation. Effect sizes are based on guidelines designed to explain the interpretation of the size of a correlation (Gignac and Szodorai 2016). These guidelines vary amongst literature, therefore the authors opted to use Pearson's *r* = 0.10, 0.20 and 0.30, to interpret the small, medium and large effect sizes (Brydges 2019; Gignac and Szodorai 2016). Dancey and Reidy (2007) was used to interpret Spearman's correlation coefficients with weak correlations ranging from 0.1 to 0.3, moderate correlations from 0.4 to 0.6 and strong correlations from 0.7 to 0.9. Stepwise multiple regression analyses were performed to investigate the contribution of certain variables to underestimation and overestimation of falls risk. The purpose of conducting multiple regression analyses was to explore the predictive ability of patient characteristics on patients' falls risk awareness. Data was screened for outliers, normality, multicollinearity and heteroscedasticity. Variables were included in the regression if there was a significant correlation with the section A SAFRM scores, which was the dependent variable. The Bonferroni correction was applied to the *p* values of significant variables.

#### 4.6.1 Factor Analysis

Establishing construct validity for the interpretations of an instrument is imperative to high quality assessment and future research outcomes (Tavakol and Wetzel 2020). This was conducted to determine if the items of section A of the SAFRM accurately represented the theoretical construct of self‐awareness. Exploratory Factor Analysis (EFA) is a multivariate technique that explores if latent, or hidden variables can describe individual or measured variables (Schreiber 2021). The factor solution derived from an EFA provides a snapshot of the statistical relationships of the key behaviours, attitudes and disposition of the construct of interest (Tavakol and Wetzel 2020). The EFA was conducted using the Jamovi software program, version 2.3.21. (The Jamovi Project 2023).

## 5 Results

### 5.1 Participant Characteristics

A total of 77 inpatients from three regional hospitals consented to be in the study (*n* = 39 from hospital 1, *n* = 23 from hospital 2 and *n* = 15 from hospital 3). Clinician responses were received from 58 health professionals, with some providing care for multiple patients. Clinicians were mostly registered nurses (*n* = 55) with responses from two physiotherapists and one occupational therapist. The average years of clinical experience amongst clinicians was 6.4 years of clinical practice with 19.5% of clinician participants characterised as graduate health professionals. The mean age of patient participants was 72.9 years (±11.2) with 62% of the sample male. The majority of patient participants identified English as their primary language (94.8%) and were born in Australia (82%). A total of 55.8% of patient participants had fallen in the past 6 months and 76.6% were classified as having a high risk for falling. Detailed participant characteristics are presented in Appendix S1. The estimated survey completion time was eight to 10 min for patients and 5 min for clinicians.

### 5.2 Objective I: Section A of SAFRM

Table 1 displays the summary section A SAFRM scores, including their classification as either complete awareness, under‐ or overestimation of falls risk compared with their clinician. The level of disparity was classified as either mild, moderate or severe. Overall, there was a difference in falls risk awareness for 42% of the sample (*n* = 32). Compared to their treating clinician, 30% of participants underestimated their falls risk (*n* = 23) and 12% overestimated their falls risk (*n* = 9). Notably, most participants who underestimated their falls risk were classified as either mild (12%) or moderate (14%) disparity in falls risk awareness. According to the SAFRM scores, 58% of participants had complete awareness of their falls risk.

**TABLE 1:** Results from Section A of the SAFRM.

Results from Section A from SAFRM (Score disparity)	*N* (%)	Average patient scores (SD)	Average clinician scores (SD)	Disparity between scores (SD)
Underestimation of falls risk (Score disparity)	23 (30%)	61.00 ± 8.5	38.57 ± 9.2	22.43 ± 8.3
Mild (11 to 20)	9 (12%)	57.78 ± 7.7	43.89 ± 7.7	13.89 ± 2.7
Moderate (21 to 30)	11 (14%)	63.45 ± 8.8	37.45 ± 7.95	26.00 ± 3.1
Severe (> 30)	3 (4%)	61.67 ± 9.1	26.67 ± 6.1	35.00 ± 6.1
Overestimation of falls risk	9 (12%)	42.67 ± 10.5	59.11 ± 12.9	−16.44 ± 6.2
Mild (−11 to −20)	7 (9%)	43.14 ± 12.1	57.0 ± 14.0	−13.86 ± 2.97
Moderate (−21 to −30)	2 (3%)	41.00 ± 2.8	66.5 ± 3.5	−25.5 ± 6.4
Severe (< −30)	0 (0%)	—	—	—
Complete Awareness of falls risk (−10 to 10)	45 (58%)	59.3 ± 13.96	60.71 ± 13.7	−0.78 ± 5.3

Abbreviation: SD, Standard deviation.

The sample size for both patient and clinician was > 50, therefore a Kolmogorov–Smirnov test of normality was conducted (Mishra et al. 2019). These results were statistically significant (*p* < 0.05), indicating that the data was not normally distributed and was suitable for non‐parametric testing. Using Wilcoxon Signed‐Ranks tests, the analysis revealed that patient SAFRM scores were higher (Md = 61.00, *n* = 77) compared to clinicians (Md = 52.00, *n* = 77), with a small to moderate effect size (*r* = 0.17). Higher patient SAFRM scores denote an underestimation of falls risk. As discussed in Mihaljcic et al. (2015), the treatment of over‐ and underestimating of falls risk as a continuum may impact the interpretability of results. Therefore, sub‐analyses of the data were conducted. To explore underestimation of falls risk, those who had overestimated their falls risk (*n* = 9) were removed from the analysis. Conversely, to explore overestimation of falls risk, participants who had underestimated their falls risk (*n* = 23) were removed. Table 2 presents the results of these analyses. In the underestimation and awareness group, patient SAFRM scores were higher (Md = 63.50, *n* = 68) compared to clinicians (Md = 52.00, *n* = 68), with a large effect size (*r* = 0.31). In the overestimation and awareness group, clinician SAFRM scores were higher (Md = 64.00, *n* = 54) than patients (Md = 60.50, *n* = 54), with a medium effect size (*r* = 0.26).

**TABLE 2:** Analysis of SAFRM scores.

SAFRM categories	*Z*	Median (Md) patient SAFRM scores	Median (Md) clinician SAFRM scores	Asymp. Sig. (2‐tailed) *p*	Effect size (*r*)
Total SAFRM scores (*n* = 77)	−2.08	61.00	52.00	0.04	0.17
Underestimation of falls risk and awareness of falls risk (*n* = 68)	−3.63	63.50	52.00	< 0.001	0.31
Overestimation of falls risk and awareness of falls risk (*n* = 54)	−2.69	60.50	64.00	0.01	0.26

Abbreviations: Asymp. Sig, Asymptotic significance; Md, median; SD, Standard deviation.

The results indicate a significant difference in patients' measures of falls risk awareness compared to that of a health professional. The large effect size reveals that patient participants were more likely to underestimate their falls risk, leading to a greater difference in median SAFRM scores between patients and clinicians.

### 5.3 Objective II: Underestimation of Falls Risk and Patient Characteristics

The relationship between underestimating falls risk and specific patient characteristics was investigated using Spearman's rho correlation coefficient. There were nine variables associated with reduced self‐awareness and are presented in Table 3.

**TABLE 3:** Associations between underestimating falls risk and patient characteristics.

Variable	Underestimation of falls risk & awareness (*n* = 68)
Age	0.39**
SMMSE	−0.33**
Anticoagulant medication use	0.35**
No gait aid required	−0.42**
PUF/4WW	0.26*
Independent mobility	−0.48**
Mobility under supervision	0.25*
One person assist with mobility	0.30*
Highest Education attained (Bachelor's)	0.25*

* Correlation is significant at the 0.05 level (2‐tailed).

** Correlation is significant at the 0.01 level (2‐tailed).

Abbreviations: PUF/4WW, pick up frame/four wheeled walker; SMMSE, Standardised Mini‐Mental State Examination.

There was a moderate correlation with underestimating falls risk and age and independent mobility. There was a weak‐moderate correlation with underestimating falls risk and anticoagulant use. Weak correlations were observed with underestimating falls risk and a Bachelor's degree, patients mobilising under supervision, patients requiring one person assist with mobility and decreased cognition. These variables were investigated using stepwise multiple regression, with the significant results detailed in Table 4.

**TABLE 4:** Regression analysis for underestimating falls risk and patient characteristics.

			95% CI			
Variable	Unstandardised Beta (*β*)	SE	LL	UL	Standardised coefficients *β*	*p*
Independent mobility	−0.644	0.208	−1.061	−0.228	−0.327	0.003
Age	0.026	0.009	0.008	0.044	0.307	0.004
Not taking anticoagulant medication	0.499	−0.200	−0.899	−0.099	−0.254	0.015

Abbreviations: CI, confidence interval; LL, lower bound of 95% confidence interval; SE, coefficients standard error; UL, upper bound of 95% confidence interval.

The regression was significant with three variables explaining 33.4% of the variance for underestimation of falls risk (*F*_3,64_ = 12.217, *p* < 0.001). There was an independent association with underestimation of falls risk and for participants who were classed as having independent mobility, were increasing in age and who were not on anticoagulation medication.

### 5.4 Objective II: Overestimation of Falls Risk and Patient Characteristics

The relationship between overestimating falls risk and specific patient characteristics was investigated using Spearman's rho correlation coefficient. The variables associated with overestimation of falls risk are presented in Table 5.

**TABLE 5:** Associations between overestimating falls risk and patient characteristics.

Variable	Overestimation of falls risk & awareness (*n* = 54)
Anticoagulant medication use	0.33**
Cultural background (Born overseas)	0.38**
Highest education attained (< year 11)	−0.32*
Highest education (postgraduate)	0.29*

* Correlation is significant at the 0.05 level (2‐tailed).

** Correlation is significant at the 0.01 level (2‐tailed).

There were weak associations between overestimating falls risk and anticoagulant medication use and education level (Postgraduate and less than year 11). A moderate association was observed between overestimating falls risk and cultural background. The overestimation model had fewer predictors but was significant, explaining 23.2% of the total variance (*F*_2,50_ = 8.841, *p* < 0.001). As presented in Table 6, patient participants were more likely to overestimate their falls risk if they were on anticoagulant medication and if their highest education level was year 11 or lower.

**TABLE 6:** Regression analysis for overestimating falls risk and patient characteristics.

Variable	95% CI					
	Unstandardised beta (*β*)	SE	LL	UL	Standardised Coefficients *β*	*p*
Anticoagulant medication use	0.528	0.176	0.174	0.882	0.364	0.004
Highest Education attained (< year 11)	−0.490	0.173	−0.837	−0.143	−0.345	0.007

Abbreviations: CI, confidence interval; LL, lower bound of 95% confidence interval; SE, coefficients standard error; UL, upper bound of 95% confidence interval.

### 5.5 Objective III: Exploratory Factor Analysis

When conducting an EFA, researchers are advised to make decisions regarding the estimation method, the number of factors to retain, the rotation method and the method for calculating scores (de Winter and Dodou 2012). Before proceeding with EFA, the Kaiser‐Meyer‐Olkin Measure of Sampling Adequacy and Bartlett's test of sphericity were used to evaluate the suitability of the dataset. Results indicate that the Kaiser‐Meyer‐Olkin measure was sufficient at 0.91, which exceeds the recommended value of 0.6 (Pallant 2011). Bartlett's test of sphericity was also statistically significant (*p* < 0.001), supporting the suitability of factor analysis (Pallant 2011). Factors were extracted by principal axis factoring method and rotated by Promax rotation. Promax is a method for oblique rotation, which means that the factors are allowed to correlate, in contrast to orthogonal rotation (Field 2013).

**FIGURE 1:**
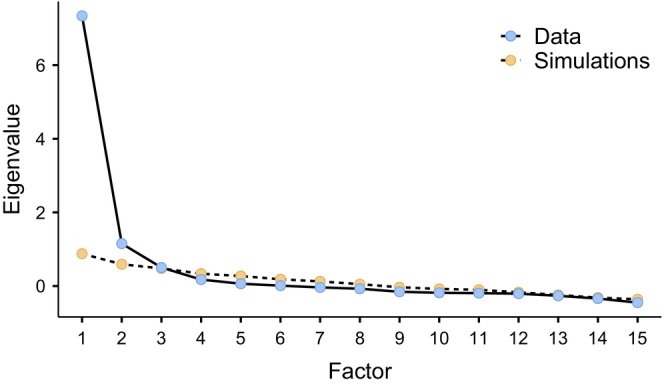
Scree plot depicting the parallel analysis.

The number of factors were decided in consideration of visual inspection of the scree plot, parallel analysis, cumulative variance explained and interpretability of the results. A total of three factors were extracted and rotated and the cumulative variance explained was 63.3%. The initial eigenvalues of these three factors were 7.33, 1.15 and 0.50. Although the third factor was 0.50, which is below Kaiser's criterion value of 1.0 (Pallant 2011), it was decided to retain this factor based on visual inspection of the scree plot (see Figure 1) and parallel analysis. Item factor loading cut‐off was set at 0.3.

The solution shows that items were grouped into three factors. These were subsequently named Physical Activity Awareness (SAFRM Items 10, 11, 12, 13, 14 and 15), Cognitive Awareness (SAFRM Items 1, 6, 7, 8 and 9) and Balance Awareness (SAFRM Items 2, 3, 4, and 5). Table 7 shows the individual factor loading for the 15‐item SAFRM. The model fit measures were RMSEA = 0.087, TLI of 0.918 and χ^2^ = 137, *df* 63, *p* < 0.001, which denotes a marginal model fit. SAFRM item 15 (standing from a chair) cross loaded on two factors (Factors 1 and 3). It was decided to retain this item in factor 1 for three reasons. Firstly, item 15 had a higher factor loading in factor 1 (0.528 compared with 0.358 in Factor 3). Removing the item from the model did not impact the model fit testing results (RMSEA = 0.085, TLI of 0.924 and χ^2^ = 110, *df* 52, *p* < 0.001). Finally, a reliability analysis showed that the Cronbach's alpha coefficient was 0.89 when removing item 15 from factor 3, thereby not affecting the internal consistency of the third factor.

**TABLE 7:** Factor loadings.

	Factor			Uniqueness
	1	2	3	
Vision		0.399		0.794
2 Balance (transfers)			0.683	0.290
3 Leg strength			0.909	0.231
4 Steadiness (walking)			0.807	0.245
5 Proprioception			0.338	0.410
6 Impulsivity		0.705		0.436
7 Concentration		0.789		0.390
8 Following instructions		0.841		0.398
9 Safety Decisions		0.658		0.534
10 Showering	0.754			0.420
11 Walking (bathroom)	0.892			0.164
12 Climbing stairs	0.650			0.337
13 Toileting	0.986			0.195
14 Walking 30 m	0.730			0.344
15 Standing from a chair	0.528			0.316

*Note:* ‘Principal axis factoring’ extraction method was used in combination with a ‘Promax’ rotation.

The results show a high unique variance for item 1 (Vision) at 0.794, which means that this variable may not be relevant to the factor model. Similarly, removing item 1 from the model did not significantly impact the model fit testing results (RMSEA = 0.092, TLI of 0.918 and χ^2^ = 121, *df* 52, *p* < 0.001). A reliability analysis showed a slight improvement in the internal consistency of Factor 2 if item 1 was dropped (Cronbach's α = 0.825 instead of 0.81), however it was decided to keep item 1 in the model due to the overall model fit.

Given the high correlation and similarities in factors between Physical Activity Awareness (Factor 1) and Balance Awareness (Factor 3) at *r* = 0.775, the authors tested a two‐factor solution. The model fit measures for the two‐factor solution were RMSEA = 0.125, TLI of 0.830 and χ^2^ = 261, *df* 76, *p* < 0.001, which denotes a poor model fit. Therefore, the authors collectively decided to retain the three‐factor solution.

### 5.6 Objective IV: Reliability Analysis

A reliability analysis was conducted to determine the internal consistency of each component. The Cronbach alpha coefficient was 0.93, which is slightly higher than the original study. With the three‐factor solution, factor 1 (Physical Activity Awareness) resulted in a Cronbach alpha coefficient of 0.93, factor 2 (Cognitive Awareness) resulted in a Cronbach alpha coefficient of 0.81 and factor 3 (Balance Awareness) resulted in a Cronbach alpha coefficient of 0.89. Table 8 details the individual Cronbach's alpha scores for each individual item if they were excluded. It was decided to retain all individual items due to the lack of overall variance to the internal consistency of the scale.

**TABLE 8:** Reliability analysis if item is dropped.

Item	Cronbach's α if item dropped		
	Factor 1	Factor 2	Factor 3
1. Vision		0.825	
2. Balance (transfers)			0.857
3. Leg Strength			0.847
4. Steadiness (walking)			0.844
5. Proprioception			0.896
6. Impulsivity		0.753	
7. Concentration		0.743	
8. Following instructions		0.755	
9. Safety decisions		0.765	
10. Showering	0.921		
11. Walking (bathroom)	0.902		
12. Climbing stairs	0.914		
13. Toileting	0.908		
14. Walking 30 m	0.914		
15. Standing from a chair	0.916		

Based on factor loading and reliability analysis, the results support a three‐factor solution for intellectual awareness of the SAFRM. These latent constructs have been labelled Physical Activity Awareness, Cognitive Awareness and Balance Awareness. Further research of this proposed model is recommended with a new, larger dataset, using confirmatory factor analysis (CFA).

## 6 Discussion

The first research objective of this study was to determine the extent of disparities in patients' falls risk awareness in hospital against that of a health professional. Based on the SAFRM scores, the findings indicated there was a difference in falls risk awareness for 42% of the sample, with 58% of participants accurately identifying their falls risk. These results differ slightly to Mihaljcic et al. (2015), in which they report accurate patient falls risk awareness in 46% of their sample, with a falls risk disparity in 54% using the first subsection of the SAFRM. These differences could have stemmed from the diverse sampling in the current study, as the original patient population in Mihaljcic et al. (2015) were recruited from a rehabilitation setting only. It is also important to note that in Mihaljcic et al. (2015), the participants were on average 5 years older and were assessed by physiotherapists. In the present study, registered nurses were the main health professionals involved in assessing patient participants, which could lead to varying levels of sensitivity towards falls risk awareness by both participants and health professionals. Similar to Mihaljcic et al. (2015), participants in the current study were more likely to underestimate their falls risk than overestimate. This is important because reduced falls risk awareness can result in poor engagement with fall prevention strategies (Mihaljcic et al. 2017; Twibell et al. 2015). The benefit of the SAFRM scoring system is the ability to differentiate between an under‐ or overestimation of falls risk and the magnitude of any disparities. This information is useful for the health professional to tailor falls prevention education and management, in collaboration with the patient.

The second research objective was to establish if a relationship existed between inpatients' falls risk disparities and patient characteristics. Similar to Mihaljcic et al. (2015), the results of the current study did not establish an association with self‐awareness of falls risk and previous falls. This was surprising given that 55.8% of participants had sustained a fall in the past 6 months. These results underscore the importance of patient education, given that a history of prior falls is a non‐modifiable risk factor for future falls (Ambrose, Paul, and Hausdorff 2013). However it cannot be concluded that education is the primary factor in reducing falls, as other unknown variables could have impacted the results. A strength of this study is the independent analysis of factors associated with underestimation and overestimation of falls risk. Underestimating falls risk was associated with age and for those identified as having independent mobility. These findings are comparable with Haines and McPhail (2011), in which older adults with a higher Functional Independence Measure Cognitive score were less likely to rate their risk of falling highly. An interesting finding of this study is the association of anticoagulant use with perceived falls risk. Participants on anticoagulant medication were more likely to overestimate their falls risk. A history of falls is not an independent predictor of bleeding for those on anticoagulants (Hindricks et al. 2021) and studies have found there is not a significantly increased risk of major bleeds for patients who are a high falls risk and on oral anticoagulation (Donzé et al. 2012). Perception of a high risk of falling should not be the deciding factor to withhold anticoagulation in older adults, as the benefits of anticoagulation outweigh the risk of bleeding (Hagerty and Rich 2017; Wei et al. 2021). Further patient education about anticoagulant use may be required, especially in those who have an increased concern for falling.

The third and fourth objectives of this study were to investigate the implementation of section A of the SAFRM to measure falls risk awareness in patients. A recent COSMIN systematic review reported a total of 20 validated falls risk perception instruments, specific to a hospital setting (Dabkowski et al. 2023). Most of these patient reported outcome measures (PROMs) received Class B recommendations according to COSMIN methodology, confirming the need for further validation studies. The SAFRM was one such measure and the fact that it measured patient and clinician perception, increased the usefulness of this instrument. The results of the EFA demonstrate the reliability and validity of the 15‐item SAFRM in measuring falls risk perceptions, however further research via CFA is recommended with a new dataset to strengthen these findings. Moreover, the identification of the three latent variables could be used to inform falls education. For example, if a clinician uses the 15‐item survey and identifies that a patient has a greater disparity in the items relating to balance awareness, they may choose to focus rehabilitation for this area. This is beneficial as a recent systematic review and meta‐analysis discussed that evidence‐based falls education can reduce falls rates in hospital (Morris et al. 2022). Further research could explore the integration and efficacy of the 15‐item SAFRM with hospital falls education.

The recent World Guidelines for Falls Prevention and Management strongly recommended to use a standardised instrument to assess falls risk perceptions and concerns in acute care hospitals or long‐term facilities (Montero‐Odasso et al. 2022). However, it is important to note that there is no ‘gold standard’ that will provide a complete falls risk assessment, given the multifactorial nature of falls (Strini, Schiavolin, and Prendin 2021). Clinical guidelines advise against using falls risk prediction tools to predict inpatients' risk of falling in hospital (National Institute for Health and Care Excellence 2013). The SAFRM offers a promising, person‐centred approach to assessing falls risk perception in hospital, which could be used to direct falls prevention education and management.

## 7 Study Limitations

A limitation to this study, is the use of the shortened‐SAFRM, which measured one level of self‐awareness (intellectual awareness). Given that the SAFRM was founded on the Self‐Awareness Model of three hierarchical levels (Crosson et al. 1989), the other two levels of emergent or anticipatory awareness were not assessed, which may limit the findings of overall self‐awareness. This was a purposeful decision by the authors with the intent to explore the shortened version of the tool as a feasible option. A further limitation to this study is the use of only one clinician per patient to rate the 15‐item SAFRM. Mihaljcic et al. (2014) recommended two clinicians to rate all sections of the SAFRM together and to settle on an average patient score. The authors opted to use one clinician, given that only 15‐items of the 31‐item SAFRM were being assessed. In the original study, section A of the SAFRM showed excellent inter‐rater reliability of 0.78 (Mihaljcic et al. 2014), which supports our decision to include one set of clinician scores per patient. Additionally, patients with severe cognition deficits were not included in this study, therefore the findings of this study may not be applicable to this population. The FRAT scores obtained from the patients' medical records were assessed by different clinicians, therefore it is possible that these scores may vary. The sampling strategy did not include the emergency departments, maternity, critical care units or specialised mental health wards. Further studies may consider the inclusion of these populations in future falls risk perception research.

Although the sample size was comparable to Mihaljcic et al. (2014), it was still relatively small, which could be viewed as a limitation, particularly when conducting EFA. Similarly, the consecutive sampling approach could be deemed a limitation. Participants were selected according to accessibility, with participant recruitment ceasing when the calculated sample size was attained. As a result, the participants may not reflect a representative sample of the population, which could have implications for the generalisability of the results. Further studies could incorporate a larger sample size.

Finally, this study was conducted over the Australian winter period, which may have further implications for the findings. Previous studies such as Magota et al. (2017) reported an increased number of falls in hospital during the winter months, compared to summer. Future research could examine if seasonal changes affect patients' falls risk awareness.

## 8 Conclusion

This study has established that the 15‐item SAFRM is a feasible and reliable falls risk perception measure for use in a hospital setting. The results confirmed previous findings by establishing a significant difference in patients' measures of falls risk awareness compared to those of health professionals, highlighting the importance of understanding these discrepancies. The ability to identify an under‐ or overestimation of falls risk in patients is useful to inform falls education and strategies. Additionally, this study found independent associations of variables with falls risk perception, which further understanding of these contributions to differences in falls risk awareness. These findings provide the opportunity to further address falls risk disparities in hospital inpatients. The results from the EFA have extended previous findings of the SAFRM structure, by identifying three latent variables for intellectual awareness. Opportunities for future research using the 15‐item SAFRM have been underscored with the recommendation to conduct confirmatory factor analysis with a large sample size.

## Author Contributions

This paper reports the findings of a PhD study. Conceptualisation: E.D., S.J.C., J.D. and K.M. Data curation: E.D. Formal analysis: E.D. Funding acquisition: S.J.C., J.D. and K.M. Investigation: E.D. Methodology: E.D., S.J.C., J.D., and K.M. Validation: E.D. Supervision: S.J.C, J.D and K.M. Writing – original draft: E.D. Writing – review and editing: S.J.C., J.D., K.M. and E.D.

## Ethics Statement

Ethical approval was received from both Latrobe Regional Health Human Research Ethics Committee (2022‐02 HREA) and Federation University Human Research Ethics Committee (2022‐057).

## Consent

All participants provided written informed consent prior to enrolment in the study.

## Conflicts of Interest

The authors declare no conflicts of interest.

## Statistical Guideline Statement

The statistics were checked prior to submission by an expert statistician, Dr. Huy Nguyen. Email: h.vannguyen@federation.edu.au.

## Supporting information


Data S1.

**Appendix S1.** Participant Characteristics.

## Data Availability

Data is available upon reasonable request from the authors.

## References

[nop270099-bib-0001] Ambrose, A. F. , G. Paul , and J. M. Hausdorff . 2013. “Risk Factors for Falls Among Older Adults: A Review of the Literature.” Maturitas 75, no. 1: 51–61. 10.1016/j.maturitas.2013.02.009. 23523272

[nop270099-bib-0002] Australian Institute of Health and Welfare . 2022. “Health Care Quality and Safety.” Retrieved on December 16, 2022. https://www.aihw.gov.au/reports/health‐care‐quality‐performance/health‐care‐safety‐and‐quality.

[nop270099-bib-0003] Black, N. 2013. “Patient Reported Outcome Measures Could Help Transform Healthcare.” British Medical Journal 346: f167. 10.1136/bmj.f167. 23358487

[nop270099-bib-0004] Brydges, C. R. 2019. “Effect Size Guidelines, Sample Size Calculations, and Statistical Power in Gerontology.” Innovation in Aging 3, no. 4: igz036. 10.1093/geroni/igz036. 31528719 PMC6736231

[nop270099-bib-0005] Caldwell, C. , and L. A. Hayes . 2016. “Self‐Efficacy and Self‐Awareness: Moral Insights to Increased Leader Effectiveness.” Journal of Management Development 35, no. 9: 1163–1173. 10.1108/JMD-01-2016-0011.

[nop270099-bib-0006] Crosson, B. , P. P. Barco , C. A. Velozo , et al. 1989. “Awareness and Compensation in Postacute Head Injury Rehabilitation.” Journal of Head Trauma Rehabilitation 4, no. 3: 46–54. 10.1097/00001199-198909000-00008.

[nop270099-bib-0007] Dabkowski, E. , S. Cooper , J. R. Duncan , and K. Missen . 2022. “Adult inpatients' Perceptions of Their Fall Risk: A Scoping Review.” Healthcare 10, no. 6: 995. 10.3390/healthcare10060995. 35742046 PMC9222288

[nop270099-bib-0008] Dabkowski, E. , S. J. Cooper , J. R. Duncan , and K. Missen . 2023. “Exploring Hospital Inpatients' Awareness of Their Falls Risk: A Qualitative Exploratory Study.” International Journal of Environmental Research and Public Health 20, no. 1: 454. 10.3390/ijerph20010454. PMC9819707 36612780

[nop270099-bib-0009] Dabkowski, E. , K. Missen , J. R. Duncan , and S. J. Cooper . 2023. “Falls Risk Perception Measures in Hospital: A COSMIN Systematic Review.” Journal of Patient Repoorted Outcomes 7: 58. 10.1186/s41687-023-00603-w. PMC10293508 37358752

[nop270099-bib-0010] Dancey, C. P. , and J. Reidy . 2007. Statistics Without Maths for Psychology. Harlow, UK: Pearson Education.

[nop270099-bib-0011] de Winter, J. C. F. , and D. Dodou . 2012. “Factor Recovery by Principal Axis Factoring and Maximum Likelihood Factor Analysis as a Function of Factor Pattern and Sample Size.” Journal of Applied Statistics 39, no. 4: 695–710. 10.1080/02664763.2011.610445.

[nop270099-bib-0012] Deandrea, S. , F. Bravi , F. Turati , E. Lucenteforte , C. La Vecchia , and E. Negri . 2013. “Risk Factors for Falls in Older People in Nursing Homes and Hospitals. A Systematic Review and Meta‐Analysis.” Archives of Gerontology and Geriatrics 56, no. 3: 407–415. 10.1016/j.archger.2012.12.006. 23294998

[nop270099-bib-0013] Dolan, H. , M. Slebodnik , and R. Taylor‐Piliae . 2021. “Older Adults' Perceptions of Their Fall Risk in the Hospital: An Integrative Review.” Journal of Clinical Nursing 31: 2418–2436. 10.1111/jocn.16125. 34786777

[nop270099-bib-0014] Donzé, J. , C. Clair , B. Hug , et al. 2012. “Risk of Falls and Major Bleeds in Patients on Oral Anticoagulation Therapy.” American Journal of Medicine 125, no. 8: 773–778. 10.1016/j.amjmed.2012.01.033. 22840664

[nop270099-bib-0015] Faul, F. , E. Erdfelder , A.‐G. Lang , and A. Buchner . 2007. “G*Power 3: A Flexible Statistical Power Analysis Program for the Social, Behavioral, and Biomedical Sciences.” Behavior Research Methods 39, no. 2: 175–191. 10.3758/BF03193146. 17695343

[nop270099-bib-0016] Field, A. 2013. Discovering Statistics Using IBM SPSS Statistics. 4th ed. London, UK: Sage Publications Ltd.

[nop270099-bib-0017] Gignac, G. E. , and E. T. Szodorai . 2016. “Effect Size Guidelines for Individual Differences Researchers.” Personality and Individual Differences 102: 74–78. 10.1016/j.paid.2016.06.069.

[nop270099-bib-0018] Glasgow, R. E. , and W. T. Riley . 2013. “Pragmatic Measures: What They Are and Why We Need Them.” American Journal of Preventive Medicine 45, no. 2: 237–243. 10.1016/j.amepre.2013.03.010. 23867032

[nop270099-bib-0019] Hagerty, T. , and M. W. Rich . 2017. “Fall Risk and Anticoagulation for Atrial Fibrillation in the Elderly: A Delicate Balance.” Cleveland Clinic Journal of Medicine 84, no. 1: 35–40. 10.3949/ccjm.84a.16016. 28084982

[nop270099-bib-0020] Haines, T. P. , and S. McPhail . 2011. “Threat Appraisal for Harm From Falls: Insights for Development of Education‐Based Intervention.” Open Longevity Science 5: 9–15.

[nop270099-bib-0021] Härlein, J. , T. Dassen , R. J. Halfens , and C. Heinze . 2009. “Fall Risk Factors in Older People With Dementia or Cognitive Impairment: A Systematic Review.” Journal of Advanced Nursing 65, no. 5: 922–933. 10.1111/j.1365-2648.2008.04950.x. 19291191

[nop270099-bib-0022] Heng, H. , D. Jazayeri , L. Shaw , D. Kiegaldie , A.‐M. Hill , and M. E. Morris . 2020. “Hospital Falls Prevention With Patient Education: A Scoping Review.” BMC Geriatrics 20, no. 1: 140. 10.1186/s12877-020-01515-w. 32293298 PMC7161005

[nop270099-bib-0023] Hindricks, G. , T. Potpara , N. Dagres , et al. 2021. “2020 ESC Guidelines for the Diagnosis and Management of Atrial Fibrillation Developed in Collaboration With the European Association for Cardio‐Thoracic Surgery (EACTS): The Task Force for the Diagnosis and Management of Atrial Fibrillation of the European Society of Cardiology (ESC) Developed With the Special Contribution of the European Heart Rhythm Association (EHRA) of the ESC.” European Heart Journal 42, no. 5: 373–498. 10.1093/eurheartj/ehaa612. 32860505

[nop270099-bib-0024] Hughes, C. C. , I. I. Kneebone , F. Jones , and B. Brady . 2015. “A Theoretical and Empirical Review of Psychological Factors Associated With Falls‐Related Psychological Concerns in Community‐Dwelling Older People.” International Psychogeriatrics 27, no. 7: 1071–1087. 10.1017/S1041610214002701. 25633917

[nop270099-bib-0025] Jørstad, E. C. , K. Hauer , C. Becker , S. E. Lamb , and ProFaNE Group . 2005. “Measuring the Psychological Outcomes of Falling: A Systematic Review.” Journal of the American Geriatrics Society 53, no. 3: 501–510. 10.1111/j.1532-5415.2005.53172.x. 15743297

[nop270099-bib-0026] LeLaurin, J. H. , and R. I. Shorr . 2019. “Preventing Falls in Hospitalized Patients: State of the Science.” Clinics in Geriatric Medicine 35, no. 2: 273–283. 10.1016/j.cger.2019.01.007. 30929888 PMC6446937

[nop270099-bib-0027] Magota, C. , H. Sawatari , S. I. Ando , et al. 2017. “Seasonal Ambient Changes Influence Inpatient Falls.” Age and Ageing 46, no. 3: 513–517. 10.1093/ageing/afw254. 28057622

[nop270099-bib-0028] Mihaljcic, T. , T. P. Haines , J. L. Ponsford , and R. J. Stolwyk . 2014. “Development of a New Self‐Awareness of Falls Risk Measure (SAFRM).” Archives of Gerontology and Geriatrics 59, no. 2: 249–256. 10.1016/j.archger.2014.06.001. 24997096

[nop270099-bib-0029] Mihaljcic, T. , T. P. Haines , J. L. Ponsford , and R. J. Stolwyk . 2015. “Self‐Awareness of Falls Risk Among Elderly Patients: Characterizing Awareness Deficits and Exploring Associated Factors.” Archives of Physical Medicine and Rehabilitation 96, no. 12: 2145–2152. 10.1016/j.apmr.2015.08.414. 26301387

[nop270099-bib-0030] Mihaljcic, T. , T. P. Haines , J. L. Ponsford , and R. J. Stolwyk . 2017. “Investigating the Relationship Between Reduced Self‐Awareness of Falls Risk, Rehabilitation Engagement and Falls in Older Adults.” Archives of Gerontology and Geriatrics 69: 38–44. 10.1016/j.archger.2016.11.003. 27886565

[nop270099-bib-0031] Mishra, P. , C. M. Pandey , U. Singh , A. Gupta , C. Sahu , and A. Keshri . 2019. “Descriptive Statistics and Normality Tests for Statistical Data.” Annals of Cardiac Anaesthesia 22, no. 1: 67–72. 10.4103/aca.ACA_157_18. 30648682 PMC6350423

[nop270099-bib-0032] Mokkink, L. B. , C. Prinsen , D. L. Patrick , et al. 2018. “COSMIN Methodology for Systematic Reviews of Patient‐Reported Outcome Measures (PROMs).” User Manual, Issue 1. https://www.cosmin.nl/wp‐content/uploads/COSMIN‐syst‐review‐for‐PROMs‐manual_version‐1_feb‐2018‐1.pdf. 10.1007/s11136-018-1798-3 PMC5891568 29435801

[nop270099-bib-0033] Molloy, D. W. , and T. I. Standish . 1997. “A Guide to the Standardized Mini‐Mental State Examination.” International Psychogeriatrics 9 Suppl 1: 87–94. 10.1017/s1041610297004754. 9447431

[nop270099-bib-0034] Montero‐Odasso, M. , N. van der Velde , F. C. Martin , et al. 2022. “World Guidelines for Falls Prevention and Management for Older Adults: A Global Initiative.” Age and Ageing 51, no. 9: afac205. 10.1093/ageing/afac205. 36178003 PMC9523684

[nop270099-bib-0035] Moore, D. S. , and R. Ellis . 2008. “Measurement of Fall‐Related Psychological Constructs Among Independent‐Living Older Adults: A Review of the Research Literature.” Aging & Mental Health 12, no. 6: 684–699. 10.1080/13607860802148855. 19023720

[nop270099-bib-0036] Morris, M. E. , K. Webster , C. Jones , et al. 2022. “Interventions to Reduce Falls in Hospitals: A Systematic Review and Meta‐Analysis.” Age and Ageing 51, no. 5: afac077. 10.1093/ageing/afac077. 35524748 PMC9078046

[nop270099-bib-0037] National Institute for Health and Care Excellence . 2013. “Falls in Older People: Assessing Risk and Prevention.” Retrieved on March 24, 2022. https://www.nice.org.uk/guidance/cg161/chapter/1‐Recommendations. 31869045

[nop270099-bib-0038] Oliver, D. , F. Daly , F. C. Martin , and M. E. T. McMurdo . 2004. “Risk Factors and Risk Assessment Tools for Falls in Hospital In‐Patients: A Systematic Review.” Age and Ageing 33, no. 2: 122–130. 10.1093/ageing/afh017. 14960426

[nop270099-bib-0039] Pallant, J. 2011. SPSS Survival Manual. 4th ed. Crows Nest, NSW, Australia: Allen & Unwin.

[nop270099-bib-0040] Schreiber, J. B. 2021. “Issues and Recommendations for Exploratory Factor Analysis and Principal Component Analysis.” Research in Social and Administrative Pharmacy 17, no. 5: 1004–1011. 10.1016/j.sapharm.2020.07.027. 33162380

[nop270099-bib-0041] Strini, V. , R. Schiavolin , and A. Prendin . 2021. “Fall Risk Assessment Scales: A Systematic Literature Review.” Nursing Reports 11, no. 2: 430–443. 10.3390/nursrep11020041. 34968219 PMC8608097

[nop270099-bib-0042] Tavakol, M. , and A. Wetzel . 2020. “Factor Analysis: A Means for Theory and Instrument Development in Support of Construct Validity.” International Journal of Medical Education 11: 245–247. 10.5116/ijme.5f96.0f4a. 33170146 PMC7883798

[nop270099-bib-0043] The Jamovi Project . 2023. “Jamovi.” (Version 2.3.21). https://www.jamovi.org/.

[nop270099-bib-0044] Twibell, R. S. , D. Siela , T. Sproat , and G. Coers . 2015. “Perceptions Related to Falls and Fall Prevention Among Hospitalized Adults.” American Journal of Critical Care: An Official Publication, American Association of Critical‐Care Nurses 24, no. 5: e78–e85. 10.4037/ajcc2015375. 26330442

[nop270099-bib-0045] von Elm, E. , D. G. Altman , M. Egger , S. J. Pocock , P. C. Gotzsche , and J. P. Vandenbroucke . 2007. “The Strengthening the Reporting of Observational Studies in Epidemiology (STROBE) Statement: Guidelines for Reporting Observational Studies.” https://www.equator‐network.org/reporting‐guidelines/strobe/. 10.1136/bmj.39335.541782.AD PMC2034723 17947786

[nop270099-bib-0046] Wei, W. , R. S. Rasu , J. J. Hernández‐Muñoz , et al. 2021. “Impact of Fall Risk and Direct Oral Anticoagulant Treatment on Quality‐Adjusted Life‐Years in Older Adults With Atrial Fibrillation: A Markov Decision Analysis.” Drugs & Aging 38, no. 8: 713–723. 10.1007/s40266-021-00870-6. 34235644

