# Supplementary material for: Investigating Falls Risk Awareness in Hospitals Using the Self‐Awareness of Falls Risk Measure (SAFRM): Empirical Research Quantitative
Source: Nurs Open. 2024 Dec 26;12(1):e70099. doi: 10.1002/nop2.70099 (PMC11670870; doi:10.1002/nop2.70099)
Supplement: Supplementary file 1 — Data S1. [file NOP2-12-e70099-s002.pdf]

MONASH University

M8 Alliance

Medicine, Nursing and Health Sciences

Self-Awareness of Falls Risk Measure (SAFRM)

School of Psychological Sciences

Tijana Mihaljcic

Terry P Haines

Jennie L Ponsford

Renerus J Stolwyk

Corresponding author:

Renerus J Stolwyk,
School of Psychological Sciences,
Monash University, Melbourne,
VIC 3800, Australia.
Email: *Rene.Stolwyk@monash.edu*
Phone: +61 3 9902 0099
Fax: +61 3 9905 3948

Self-Awareness of Falls Risk Measure (SAFRM)

Background

The SAFRM is a 31-item scale designed to measure personal self-awareness of falls risk in adults aged over 60 years currently undergoing inpatient rehabilitation. It comprises three subscales measuring intellectual, emergent and anticipatory awareness. The intellectual subscale (15 items) measures the individual's awareness of physical, sensory and cognitive deficits which increase falls risk, and awareness of activities which increase falls risk. The emergent awareness subsection (6 items) measures the individual's ability to accurately appraise the quality of his/her walking performance, and the anticipatory awareness subsection (10 items) measures the individual's awareness of the amount of assistance he/she needs to complete activities safely in the immediate future. The SAFRM consists of two parallel forms: one form is completed by the patient and the other by the treating clinician/s. Self-awareness is conceptualised as the discrepancy between the patient and clinician-ratings.

For further information regarding the SAFRM development and validation see Mihaljcic, Ponsford, Haines and Stolwyk (2014) Developing a new self-awareness of falls risk measure (SAFRM). Archives of Gerontology and Geriatrics. 59 (2), 249-256.

Administration

The intellectual (Form I) and anticipatory (Form A) subsections are administered at time and place of convenience. The patient is provided with the patient forms (SAFRM-Patient) and asked to answer the questionnaires based on current level of ability. The treating clinician/s are provided with the clinician forms (SAFRM-Clinician) and required to answer the questionnaires based on the patient's current level of ability. **The patient and clinician forms should be completed on the same day and no later than seven days apart. The forms should be completed separately with no discussion between patients and clinicians until the forms have been completed.**

It is highly recommended that two clinicians working with the patient complete the SAFRM-Clinician together setting on an average score for each item, and that clinicians have a minimum of five days contact with the patient prior to completing the SAFRM.

The emergent (Form E) subsection is completed during standardised administration of the Timed Up-and-Go test (TUG) by a trained clinician. Immediately following the TUG, the patient is provided with Form E (SAFRM-Patient) and asked to rate his/her performance in comparison to an 'average healthy person of the same age'. As the patient is completing their form, the clinician completes Form E (SAFRM-Clinician). **Again it is important that there is no discussion between patient and clinician until the forms have been completed.**

Scoring

Items within all three subsections (Intellectual/Emergent/Anticipatory) are added to obtain section subscores. A total score is then calculated by adding section subscores. Self-awareness is calculated by subtracting the clinician ratings on the desired section (i.e. intellectual, emergent, anticipatory or total) from patient ratings on the respective section. For example,

Intellectual Self-Awareness

=

Form I Total (Patient)

–

Form I Total (Clinician)

Interpretation

A score of zero indicates perfect agreement between patient and clinician (i.e. good self-awareness). Larger discrepancy scores indicate greater disagreement between patient and clinician, and represent the patient's level of impaired self-awareness. A positive score indicates that the patient is underestimating falls risk and overestimating ability, and a negative score means that the patient is overestimating falls risk and underestimating ability.

Preliminary cut-off values have been proposed for the interpretation of the SAFRM scores, including the categorisation of mild, moderate and severe underestimation and overestimation of falls risk (see Table). Positive values within the provided mild, moderate and severe ranges correspond to reduced self-awareness (i.e. individuals that underestimate falls risk), and negative values correspond to individuals that overestimate falls risk.

	Aware	Mild	Moderate	Severe
Intellectual awareness	±10	11-20	21-30	>30
Emergent awareness	±3	4-6	7-9	>9
Anticipatory awareness	±9	10-18	19-27	>27
SAFRM total	±16	17-32	33-48	>48

Mihaljcic, Haines, Ponsford and Stolwyk (manuscript submitted).

SAFRM – Patient

Form I

Name: ______________________________ Date: ______________

Think about your current level of ability. Please circle how much each of the following factors or activities increase your risk of falling.

**1. Vision**	
5	I have **no** visual difficulties
4	I have visual difficulties, but they do not increase my risk of falling
3	My visual difficulties **mildly** increase my risk of falling
2	My visual difficulties **moderately** increase my risk of falling
1	My visual difficulties **greatly** increase my risk of falling

**2. Balance while transferring (i.e. moving from chair to bed)**	
5	I have **no** difficulties with my balance when transferring
4	I have difficulties with my balance when transferring, but this does not increase my risk of falling
3	My difficulties with balance when transferring **mildly** increases my risk of falling
2	My difficulties with balance when transferring **moderately** increases my risk of falling
1	My difficulties with balance when transferring **greatly** increases my risk of falling

**3. Leg strength**	
5	I have **no** difficulties with my leg strength
4	I have poor leg strength, but this does not increase my risk of falling
3	My poor leg strength **mildly** increases my risk of falling
2	My poor leg strength **moderately** increases my risk of falling
1	My poor leg strength **greatly** increases my risk of falling

**4. Steadiness while walking**	
5	I have **no** difficulties with my steadiness while walking
4	I have difficulties with my steadiness while walking, but this does not increase my risk of falling
3	My difficulties with steadiness while walking **mildly** increases my risk of falling
2	My difficulties with steadiness while walking **moderately** increases my risk of falling
1	My difficulties with steadiness while walking **greatly** increases my risk of falling

5. Knowing the position of your feet while walking (i.e. do you look at your feet when walking)	
5	I have **no** difficulties knowing the position of my feet while walking
4	I have difficulties knowing the position of my feet while walking, but this does not increase my risk of falling
3	My difficulties with knowing the position of my feet while walking **mildly** increases my risk of falling
2	My difficulties with knowing the position of my feet while walking **moderately** increases my risk of falling
1	My difficulties with knowing the position of my feet while walking **greatly** increases my risk of falling

6. Tendency to forget to think about your safety before you start an activity (i.e. be impulsive).	
5	I tend to think about my safety before I start an activity
4	I have a tendency to forget to think about my safety before I start an activity, but this does not increase my risk of falling
3	My tendency to forget to think about my safety before I start an activity **mildly** increases my risk of falling
2	My tendency to forget to think about my safety before I start an activity **moderately** increases my risk of falling
1	My tendency to forget to think about my safety before I start an activity **greatly** increases my risk of falling

7. Concentration	
5	I have **no** difficulties concentrating
4	I have difficulties concentrating, but this does not increase my risk of falling
3	My difficulties concentrating **mildly** increases my risk of falling
2	My difficulties concentrating **moderately** increases my risk of falling
1	My difficulties concentrating **greatly** increases my risk of falling

8. Following instructions	
5	I have **no** difficulties following instructions
4	I have difficulties following instructions, but this does not increase my risk of falling
3	My difficulties following instructions **mildly** increases my risk of falling
2	My difficulties following instructions **moderately** increases my risk of falling
1	My difficulties following instructions **greatly** increases my risk of falling

9. Deciding which activities are safe	
5	I have **no** difficulties deciding which activities are safe
4	I have difficulties deciding which activities are safe, but this does not increase my risk of falling
3	My difficulties deciding which activities are safe **mildly** increases my risk of falling
2	My difficulties deciding which activities are safe **moderately** increases my risk of falling
1	My difficulties deciding which activities are safe **greatly** increases my risk of falling

10. Showering without assistance (i.e. supervision, prompts or physical assistance) from other people	
5	I have **no** difficulties showering
4	I have difficulties showering, but this does not increase my risk of falling
3	My difficulties showering **mildly** increases my risk of falling
2	My difficulties showering **moderately** increases my risk of falling
1	My difficulties showering **greatly** increases my risk of falling

11. Walking to the bathroom without assistance (i.e. supervision, prompts or physical assistance) from other people	
5	I have **no** difficulties walking to the bathroom
4	I have difficulties walking to the bathroom, but this does not increase my risk of falling
3	My difficulties walking to the bathroom **mildly** increases my risk of falling
2	My difficulties walking to the bathroom **moderately** increases my risk of falling
1	My difficulties walking to the bathroom **greatly** increases my risk of falling

12. Climbing stairs without assistance (i.e. supervision, prompts or physical assistance) from other people	
5	I have **no** difficulties climbing stairs
4	I have difficulties climbing stairs, but this does not increase my risk of falling
3	My difficulties climbing stairs **mildly** increases my risk of falling
2	My difficulties climbing stairs **moderately** increases my risk of falling
1	My difficulties climbing stairs **greatly** increases my risk of falling

13. Using the toilet without assistance (i.e. supervision, prompts or physical assistance) from other people	
5	I have **no** difficulties using the toilet
4	I have difficulties using the toilet, but this does not increase my risk of falling
3	My difficulties using the toilet **mildly** increases my risk of falling
2	My difficulties using the toilet **moderately** increases my risk of falling
1	My difficulties using the toilet **greatly** increases my risk of falling

**14. Walking long distances (more than 30 meters) without assistance (i.e. supervision, prompts or physical assistance) from other people**	
5	I have **no** difficulties walking long distances
4	I have difficulties walking long distances, but this does not increase my risk of falling
3	My difficulties walking long distances **mildly** increases my risk of falling
2	My difficulties walking long distances **moderately** increases my risk of falling
1	My difficulties walking long distances **greatly** increases my risk of falling

**15. Standing up from a chair without assistance (i.e. supervision, prompts or physical assistance) from other people**	
5	I have **no** difficulties standing up from a chair
4	I have difficulties standing up from a chair, but this does not increase my risk of falling
3	My difficulties standing up from a chair **mildly** increases my risk of falling
2	My difficulties standing up from a chair **moderately** increases my risk of falling
1	My difficulties standing up from a chair **greatly** increases my risk of falling

Office Use Only

☐	Total Page 1
☐	Total Page 2
☐	Total Page 3
☐	Total Page 4
☐	**TOTAL Form I (Patient)**

SAFRM – Patient

Form E

Name: ______________________________ **Date:** ______________

Now that you have completed the walking task, please compare your performance on the task to that of **an average healthy person your age.**

Based on the walking task you just completed; how would you rate your:

**1. Overall performance in the task**	
5	Much Better
4	Better
3	About the Same
2	Worse
1	Much Worse

**2. Steadiness when walking**	
5	Much Better
4	Better
3	About the Same
2	Worse
1	Much Worse

**3. Steadiness when turning around**	
5	Much Better
4	Better
3	About the Same
2	Worse
1	Much Worse

**4. Ability to make continuous, equal length steps**	
5	Much Better
4	Better
3	About the Same
2	Worse
1	Much Worse

5. Ability to maintain a walking speed that is comparable to an average healthy person my age	
5	Much Better
4	Better
3	About the Same
2	Worse
1	Much Worse

6. Ability to lift your feet so they clear the floor	
5	Much Better
4	Better
3	About the Same
2	Worse
1	Much Worse

Office Use Only

☐ Total Page 1

☐ Total Page 2

☐ **TOTAL Form E (Patient)**

SAFRM – Patient

Form A

Name: ______________________________ **Date:** ______________

For each of the following activities please tick the box which best describes the level of help you would need to do the activity.

If you currently do not do the activity, please indicate the amount of assistance you would need **IF** you tried to do the activity.

1. Standing up from a chair	
7	I could do this on my own
6	I could do this on my own, but I need to use a device (e.g. mobility aid, arm rests)
5	I could do this on my own, but its good if someone watches just in case
4	I need a little bit of help
3	I need some help
2	I need a lot of help
1	I could **not** do this at all

2. Taking a shower	
7	I could do this on my own
6	I could do this on my own, but I need to use a device (e.g. mobility aid, arm rests)
5	I could do this on my own, but its good if someone watches just in case
4	I need a little bit of help
3	I need some help
2	I need a lot of help
1	I could **not** do this at all

3. Getting dressed	
7	I could do this on my own
6	I could do this on my own, but I need to use a device (e.g. mobility aid, arm rests)
5	I could do this on my own, but its good if someone watches just in case
4	I need a little bit of help
3	I need some help
2	I need a lot of help
1	I could **not** do this at all

4. Walking around your bedside	
7	I could do this on my own
6	I could do this on my own, but I need to use a device (e.g. mobility aid, arm rests)
5	I could do this on my own, but its good if someone watches just in case
4	I need a little bit of help
3	I need some help
2	I need a lot of help
1	I could **not** do this at all

5. Walking to the bathroom	
7	I could do this on my own
6	I could do this on my own, but I need to use a device (e.g. mobility aid, arm rests)
5	I could do this on my own, but its good if someone watches just in case
4	I need a little bit of help
3	I need some help
2	I need a lot of help
1	I could **not** do this at all

6. Using the toilet	
7	I could do this on my own
6	I could do this on my own, but I need to use a device (e.g. mobility aid, arm rests)
5	I could do this on my own, but its good if someone watches just in case
4	I need a little bit of help
3	I need some help
2	I need a lot of help
1	I could **not** do this at all

7. Climbing stairs	
7	I could do this on my own
6	I could do this on my own, but I need to use a device (e.g. mobility aid, arm rests)
5	I could do this on my own, but its good if someone watches just in case
4	I need a little bit of help
3	I need some help
2	I need a lot of help
1	I could **not** do this at all

8. Getting out of bed	
7	I could do this on my own
6	I could do this on my own, but I need to use a device (e.g. mobility aid, arm rests)
5	I could do this on my own, but its good if someone watches just in case
4	I need a little bit of help
3	I need some help
2	I need a lot of help
1	I could **not** do this at all

9. Getting in and out of a car	
7	I could do this on my own
6	I could do this on my own, but I need to use a device (e.g. mobility aid, arm rests)
5	I could do this on my own, but its good if someone watches just in case
4	I need a little bit of help
3	I need some help
2	I need a lot of help
1	I could **not** do this at all

10. Walking outside my dwelling (e.g. to a park, shops, bus)	
7	I could do this on my own
6	I could do this on my own, but I need to use a device (e.g. mobility aid, arm rests)
5	I could do this on my own, but its good if someone watches just in case
4	I need a little bit of help
3	I need some help
2	I need a lot of help
1	I could **not** do this at all

Office Use Only

☐	Total Page 1
☐	Total Page 2
☐	Total Page 3
☐	**TOTAL Form A (Patient)**

SAFRM – Clinician

Form I

Name: ______________________________ Date: ______________

Think about the patient's current level of ability. Please indicate how much each of the following factors or activities increase his/her risk of falling.

**1. Vision**	
5	The patient has **no** visual difficulties
4	The patient has visual difficulties, but they do not increase his/her risk of falling
3	The patient's visual difficulties **mildly** increase his/her risk of falling
2	The patient's visual difficulties **moderately** increase his/her risk of falling
1	The patient's visual difficulties **greatly** increase his/her risk of falling

**2. Balance while transferring (i.e. moving from chair to bed)**	
5	The patient has **no** difficulties with his/her balance when transferring
4	The patient has difficulties with his/her balance when transferring, but this does not increase his/her risk of falling
3	The patient's difficulties with balance when transferring **mildly** increases his/her risk of falling
2	The patient's difficulties with balance when transferring **moderately** increases his/her risk of falling
1	The patient's difficulties with balance when transferring **greatly** increases his/her risk of falling

**3. Leg strength**	
5	The patient has **no** difficulties with his/her leg strength
4	The patient has poor leg strength, but this does not increase his/her risk of falling
3	The patient's poor leg strength **mildly** increases his/her risk of falling
2	The patient's poor leg strength **moderately** increases his/her risk of falling
1	The patient's poor leg strength **greatly** increases his/her risk of falling

**4. Steadiness while walking**	
5	The patient has **no** difficulties with his/her steadiness while walking
4	The patient has difficulties with his/her steadiness while walking, but this does not increase his/her risk of falling
3	The patient's difficulties with steadiness while walking **mildly** increases his/her risk of falling
2	The patient's difficulties with steadiness while walking **moderately** increases his/her risk of falling
1	The patient's difficulties with steadiness while walking **greatly** increases his/her risk of falling

5. Knowing the position of his/her feet while walking (i.e. does the patient look at his/her feet when walking)	
5	The patient has **no** difficulties knowing the position of his/her feet while walking
4	The patient has difficulties knowing the position of his/her feet while walking, but this does not increase his/her risk of falling
3	The patient's difficulties with knowing the position of his/her feet while walking **mildly** increases his/her risk of falling
2	The patient's difficulties with knowing the position of his/her feet while walking **moderately** increases his/her risk of falling
1	The patient's difficulties with knowing the position of his/her feet while walking **greatly** increases his/her risk of falling

6. Tendency to forget to think about his/her safety before he/she starts an activity (i.e. be impulsive).	
5	The patient tends to think about his/her safety before he/she starts an activity
4	The patient has a tendency to forget to think about his/her safety before he/she starts an activity, but this does not increase his/her risk of falling
3	The patient's tendency to forget to think about his/her safety before he/she starts an activity **mildly** increases his/her risk of falling
2	The patient's tendency to forget to think about his/her safety before he/she starts an activity **moderately** increases his/her risk of falling
1	The patient's tendency to forget to think about his/her safety before he/she starts an activity **greatly** increases his/her risk of falling

7. Concentration	
5	The patient has **no** difficulties concentrating
4	The patient has difficulties concentrating, but this does not increase his/her risk of falling
3	The patient's difficulties concentrating **mildly** increases his/her risk of falling
2	The patient's difficulties concentrating **moderately** increases his/her risk of falling
1	The patient's difficulties concentrating **greatly** increases his/her risk of falling

8. Following instructions	
5	The patient has **no** difficulties following instructions
4	The patient has difficulties following instructions, but this does not increase his/her risk of falling
3	The patient's difficulties following instructions **mildly** increases his/her risk of falling
2	The patient's difficulties following instructions **moderately** increases his/her risk of falling
1	The patient's difficulties following instructions **greatly** increases his/her risk of falling

9. Deciding which activities are safe	
5	The patient has **no** difficulties deciding which activities are safe
4	The patient has difficulties deciding which activities are safe, but this does not increase his/her risk of falling
3	The patient's difficulties deciding which activities are safe **mildly** increases his/her risk of falling
2	The patient's difficulties deciding which activities are safe **moderately** increases his/her risk of falling
1	The patient's difficulties deciding which activities are safe **greatly** increases his/her risk of falling

10. Showering without assistance (i.e. supervision, prompts or physical assistance) from other people	
5	The patient has **no** difficulties showering
4	The patient has difficulties showering, but this does not increase his/her risk of falling
3	The patient's difficulties showering **mildly** increases his/her risk of falling
2	The patient's difficulties showering **moderately** increases his/her risk of falling
1	The patient's difficulties showering **greatly** increases his/her risk of falling

11. Walking to the bathroom without assistance (i.e. supervision, prompts or physical assistance) from other people	
5	The patient has **no** difficulties walking to the bathroom
4	The patient has difficulties walking to the bathroom, but this does not increase his/her risk of falling
3	The patient's difficulties walking to the bathroom **mildly** increases his/her risk of falling
2	The patient's difficulties walking to the bathroom **moderately** increases his/her risk of falling
1	The patient's difficulties walking to the bathroom **greatly** increases his/her risk of falling

12. Climbing stairs without assistance (i.e. supervision, prompts or physical assistance) from other people	
5	The patient has **no** difficulties climbing stairs
4	The patient has difficulties climbing stairs, but this does not increase his/her risk of falling
3	The patient's difficulties climbing stairs **mildly** increases his/her risk of falling
2	The patient's difficulties climbing stairs **moderately** increases his/her risk of falling
1	The patient's difficulties climbing stairs **greatly** increases his/her risk of falling

13. Using the toilet without assistance (i.e. supervision, prompts or physical assistance) from other people	
5	The patient has **no** difficulties using the toilet
4	The patient has difficulties using the toilet, but this does not increase his/her risk of falling
3	The patient's difficulties using the toilet **mildly** increases his/her risk of falling
2	The patient's difficulties using the toilet **moderately** increases his/her risk of falling
1	The patient's difficulties using the toilet **greatly** increases his/her risk of falling

14. Walking long distances (more than 30 meters) without assistance (i.e. supervision, prompts or physical assistance) from other people

5	The patient has **no** difficulties walking long distances
4	The patient has difficulties walking long distances, but this does not increase his/her risk of falling
3	The patient's difficulties walking long distances **mildly** increases his/her risk of falling
2	The patient's difficulties walking long distances **moderately** increases his/her risk of falling
1	The patient's difficulties walking long distances **greatly** increases his/her risk of falling

15. Standing up from a chair without assistance (i.e. supervision, prompts or physical assistance) from other people

5	The patient has **no** difficulties standing up from a chair
4	The patient has difficulties standing up from a chair, but it does not increase his/her risk of falling
3	The patient's difficulties standing up from a chair **mildly** increases his/her risk of falling
2	The patient's difficulties standing up from a chair **moderately** increases his/her risk of falling
1	The patient's difficulties standing up from a chair **greatly** increases his/her risk of falling

Office Use Only

☐	Total Page 1
☐	Total Page 2
☐	Total Page 3
☐	Total Page 4
☐	**TOTAL Form I (Clinican)**

Intellectual Awareness

☐ – ☐ = ☐

TOTAL Form I (Patient) – **TOTAL Form I (Clinician)** = **Intellectual Awareness**

SAFRM – Clinician

Form E

Name: ______________________________ **Date:** ______________

TUG time: ______________________ **Steps to turn:** ______________________

Now that the patient has completed the walking task, please compare his/her performance on the task to that of **an average healthy person his/her age.**

Based on the walking task the patient just completed; how would you rate his/her:

**1. Overall performance in the task**	
5	Much Better
4	Better
3	About the Same
2	Worse
1	Much Worse

**2. Steadiness when walking**	
5	Much Better
4	Better
3	About the Same
2	Worse
1	Much Worse

**3. Steadiness when turning around**	
5	Much Better
4	Better
3	About the Same
2	Worse
1	Much Worse

**4. Ability to make continuous, equal length steps**	
5	Much Better
4	Better
3	About the Same
2	Worse
1	Much Worse

5. Ability to maintain a walking speed that is comparable to an average healthy person my age	
5	Much Better
4	Better
3	About the Same
2	Worse
1	Much Worse

6. Ability to lift your feet so they clear the floor	
5	Much Better
4	Better
3	About the Same
2	Worse
1	Much Worse

Office Use Only

☐ Total Page 1

☐ Total Page 2

☐ **TOTAL Form E (Clinician)**

Emergent Awareness

☐ – ☐ = ☐

TOTAL Form E (Patient) – **TOTAL Form E (Clinician)** = **Emergent Awareness**

SAFRM – Clinician

Form A

Name: ______________________________ **Date:** ______________

For each of the following activities please tick the box which best describes the level of help the patient would need to do the activity.

If the patient currently does not do the activity, please indicate the amount of assistance the patient would need **IF** they tried to do the activity.

**1. Standing up from a chair**	
7	The patient could do this on their own
6	The patient could do this on their own, but they need to use a device (e.g. mobility aid, arm rests)
5	The patient could do this on their own, but its good if someone watches just in case
4	The patient needs a little bit of help
3	The patient needs some help
2	The patient needs a lot of help
1	The patient could **not** do this at all

**2. Taking a shower**	
7	The patient could do this on their own
6	The patient could do this on their own, but they need to use a device (e.g. mobility aid, arm rests)
5	The patient could do this on their own, but its good if someone watches just in case
4	The patient needs a little bit of help
3	The patient needs some help
2	The patient needs a lot of help
1	The patient could **not** do this at all

**3. Getting dressed**	
7	The patient could do this on their own
6	The patient could do this on their own, but they need to use a device (e.g. mobility aid, arm rests)
5	The patient could do this on their own, but its good if someone watches just in case
4	The patient needs a little bit of help
3	The patient needs some help
2	The patient needs a lot of help
1	The patient could **not** do this at all

4. Walking around his/her bedside	
7	The patient could do this on their own
6	The patient could do this on their own, but they need to use a device (e.g. mobility aid, arm rests)
5	The patient could do this on their own, but its good if someone watches just in case
4	The patient needs a little bit of help
3	The patient needs some help
2	The patient needs a lot of help
1	The patient could **not** do this at all

5. Walking to the bathroom	
7	The patient could do this on their own
6	The patient could do this on their own, but they need to use a device (e.g. mobility aid, arm rests)
5	The patient could do this on their own, but its good if someone watches just in case
4	The patient needs a little bit of help
3	The patient needs some help
2	The patient needs a lot of help
1	The patient could **not** do this at all

6. Using the toilet	
7	The patient could do this on their own
6	The patient could do this on their own, but they need to use a device (e.g. mobility aid, arm rests)
5	The patient could do this on their own, but its good if someone watches just in case
4	The patient needs a little bit of help
3	The patient needs some help
2	The patient needs a lot of help
1	The patient could **not** do this at all

7. Climbing stairs	
7	The patient could do this on their own
6	The patient could do this on their own, but they need to use a device (e.g. mobility aid, arm rests)
5	The patient could do this on their own, but its good if someone watches just in case
4	The patient needs a little bit of help
3	The patient needs some help
2	The patient needs a lot of help
1	The patient could **not** do this at all

8. Getting out of bed	
7	The patient could do this on their own
6	The patient could do this on their own, but they need to use a device (e.g. mobility aid, arm rests)
5	The patient could do this on their own, but its good if someone watches just in case
4	The patient needs a little bit of help
3	The patient needs some help
2	The patient needs a lot of help
1	The patient could **not** do this at all

9. Getting in and out of a car	
7	The patient could do this on their own
6	The patient could do this on their own, but they need to use a device (e.g. mobility aid, arm rests)
5	The patient could do this on their own, but its good if someone watches just in case
4	The patient needs a little bit of help
3	The patient needs some help
2	The patient needs a lot of help
1	The patient could **not** do this at all

10. Walking outside his/her dwelling (e.g. to a park, shops, bus)	
7	The patient could do this on their own
6	The patient could do this on their own, but they need to use a device (e.g. mobility aid, arm rests)
5	The patient could do this on their own, but its good if someone watches just in case
4	The patient needs a little bit of help
3	The patient needs some help
2	The patient needs a lot of help
1	The patient could **not** do this at all

Office Use Only

☐	Total Page 1
☐	Total Page 2
☐	Total Page 3
☐	**TOTAL Form A (Clinician)**

Anticipatory Awareness

☐	−	☐	=	☐
TOTAL Form A (Patient)		TOTAL Form A (Clinician)		Anticipatory Awareness

Overall Awareness (SAFRM Total)

☐	+	☐	+	☐	=	☐
Intellectual Awareness		Emergent Awareness		Anticipatory Awareness		SAFRM Total

Positive scores indicate that the patient is underestimating falls risk and negative scores indicate that the patient is overestimating falls risk. A score of zero represents perfect agreement (i.e. good self-awareness). For more detailed interpretation guidelines see page (i) of the administration instructions.

MONASH University

GROUP OF EIGHT AUSTRALIA

School of Psychological Sciences

Tel: +61 3 9905 3968
Fax: +61 3 9905 3948
Email: enquiries.psych@monash.edu
www.med.monash.edu/psych

CRICOS provider: Monash University 00008C. MMS374821
