# Supplementary material for: Investigating Falls Risk Awareness in Hospitals Using the Self‐Awareness of Falls Risk Measure (SAFRM): Empirical Research Quantitative
Source: Nurs Open. 2024 Dec 26;12(1):e70099. doi: 10.1002/nop2.70099 (PMC11670870; doi:10.1002/nop2.70099)
Supplement: Supplementary file 2 — Appendix S1. Participant Characteristics. [file NOP2-12-e70099-s001.docx]

**Appendix I: Participant Characteristics**

| **Variable** | **N (%)** | **Mean (SD)** | **Range** |
| --- | --- | --- | --- |
| Age (years) |  | 72.9 (11.2) | 41-98 |
| Gender  Male  Female | 48 (62%)  29 (38%) |  |  |
| Living situation  Spouse/Partner  Family  Lives alone  RACF  Independent living | 36 (46.8%)  16 (20.8%)  22 (28.6%)  1 (1.3%)  2 (2.6%) |  |  |
| Education Level  < Year 11/Certificate I/II  Year 12  Certificate III/IV  Advanced Diploma  Bachelor’s Degree  Grad. Diploma/Certificate  Postgraduate Degree  Unknown | 49 (63.6%)  8 (10.4%)  8 (10.4%)  2 (2.6%)  7 (9.1%)  1 (1.3%)  1 (1.3%)  1 (1.3%) |  |  |
| Days in hospital at data collection |  | 9.58 (18.2) | 1-119 |
| SMMSE |  | 27.6 (2.5) | 20-30 |
| Admission Diagnosis  Fall (neurological)  Fall (orthopaedic)  Fall (cardiothoracic)  Orthopaedic (not from a fall)  Medical (not from a fall)  Falls for Investigation | 5 (6.5%)  13 (16.9%)  4 (5.2%)  9 (11.7%)  40 (51.9%)  6 (7.8%) |  |  |
| Falls History in past six months  Yes  No | 43 (55.8%)  34 (44.2%) |  |  |
| Falls Risk  High  Medium  Low  Unknown | 59 (76.6%)  0 (0.0%)  11 (14.3%)  7 (9.1%) |  |  |
| Level of Supervision  Independent  Supervision  1 person assist  2 people assist | 36 (46.8%)  20 (26.0%)  15 (19.5%)  6 (7.8%) |  |  |
| Preadmission Gait Aid Use  None  4WW/PUF  SPS  Mixed | 38 (49.3%)  25 (32.5%)  8 (10.4%)  6 (7.8%) |  |  |
| Current Gait Aid Use  None  4WW/PUF  Gutter Frame  Hoist/WC  FAC  SPS | 24 (31.2%)  36 (46.8%)  2 (2.6%)  7 (9.1%)  3 (3.9%)  5 (6.5%) |  |  |
| No. of Medications at time of interview |  | 9.7 (4.63) | 1-29 |

**Abbreviations:** **FAC:** Forearm Crutches; **N:** Number; **PUF:** Pick Up Frame; **RACF:** Residential Aged Care Facility; **SMMSE:** Standardised Mini-Mental State Examination; **SPS:** Single Point Stick; **WC:** Wheelchair
